# Zinc deficiency deteriorates ovarian follicle development and function by inhibiting mitochondrial function

**DOI:** 10.1186/s13048-024-01442-z

**Published:** 2024-05-28

**Authors:** Wen-Jiao Liu, Li-Shu Li, Meng-Fan Lan, Jian-Zhou Shang, Jin-Xin Zhang, Wen-Jie Xiong, Xin-Le Lai, Xing Duan

**Affiliations:** Key Laboratory of Applied Technology on Green-Eco-Healthy Animal Husbandry of Zhejiang Province, College of Animal Science and Technology & College of Veterinary Medicine, Zhejiang A&F University, Hangzhou, Zhejiang 311300 China

**Keywords:** Zinc deficiency, Oocyte maturation, Mitochondria, Autophagy, Apoptosis

## Abstract

Zinc (Zn) is a crucial trace element essential for human growth and development, particularly for reproductive health. Previous research has shown a decrease in serum zinc concentration with age and individuals with conditions such as polycystic ovary syndrome (PCOS) and diabetes mellitus. However, the specific effects of zinc deficiency on the female reproductive system, especially ovarian function, are not fully understood. In our study, we observed a significant reduction in the total number of follicles and mature follicles in the zinc deficiency group. This reduction correlated with decreased level of anti-Mullerian hormone (AMH) and abnormal gene expression affecting hormone secretion regulation. Furthermore, we found that zinc deficiency disrupted mitochondrial dynamics, leading to oxidative stress in the ovaries, which further inhibited autophagy and increased ovarian apoptosis. These changes ultimately resulted in the failure of germinal vesicle breakdown (GVBD) and reduced oocyte quality. Meanwhile, administration of zinc glycine effectively alleviated the oocyte meiotic arrest caused by dietary zinc deficiency. In conclusion, our findings demonstrated that dietary zinc deficiency can affect hormone secretion and follicle maturation by impairing mitochondrial function and autophagy.

**Supplementary Information:**

The online version contains supplementary material available at 10.1186/s13048-024-01442-z.

## Introduction

Zinc (Zn) is an essential micronutrient present in various tissues and organs of the human body. It plays a vital role in the structure and function of numerous proteins, including transcription factors, receptors, and enzymes [1]. Zn is also involved in maintaining the intracellular redox balance, regulating the endocrine and immune systems, and promoting cell proliferation [2, 3]. Studies have shown that serum Zn concentration tends to decrease gradually with aging. This decline in Zn level could cause various health issues, including cognitive impairment and memory deficits [4]. Additionally, research has shown that the individuals with polycystic ovary syndrome (PCOS) and endometriosis usually exhibit reduced serum concentration of Zn, indicating a potential association between low zinc levels and reproductive pathologies [5–7].

Considering the fundamental importance of Zn in the control of cellular growth and differentiation, Zn has been demonstrated to play critical role in the reproductive system of both males and females. Previous studies have shown that Zn is involved maintaining the homeostasis of testosterone and regulating the sperm quality, including sperm motility, morphology, viability. Zinc deficiency in males lead to various negative effects, such as sperm DNA fragmentation, damaged sperm membranes, apoptosis, poor sperm quality, and male infertility [8, 9]. Similarly, Zn deficiency during the female early development can have adverse effect on ovary structure and function. Zn deficiency affects the concentration of progesterone, follicle-stimulating hormone (FSH) and androgen, which can lead to the failure of follicle maturation and oocyte meiosis [10, 11]. Additionally, Zn supplementation has been found to improve PCOS symptoms, particularly in women with dysregulated insulin resistance, lipid and redox balance [12].

The ovarian follicles serve as the fundamental structural and functional components of the mammalian ovary and play a crucial role in reproductive health. Comprised of oocytes, granulosa cells, and theca cells, follicles provide a supportive environment for the maturation of oocytes, which is regulated by surrounding somatic cells [13]. Throughout the female fertile lifespan, follicle development is a sequential process that begins with the recruitment of primary follicles from the primordial follicle pool, followed by growth and development of follicles, selection of a dominant follicle, and culminating in ovulation and the formation of the corpus luteum [14]. Follicular atresia, a hormone-regulated apoptotic process, naturally occurs in granulosa cells at various stages of follicular development [15]. However, dietary Zn deficiency has been shown to cause increased atresia, degeneration of granulosa follicle cells, and disruption of coronal radiation [16]. Research has shown that adding Zn to maturation medium can increase the percentage of normal morphologically follicles as the increased of Zn concentration [17]. However, the specific mechanisms through which Zn regulates follicle development have not yet been fully clarified.

Autophagy, a lysosomal-dependent self-degradation process, is required for the degradation and recycling of dysfunctional cellular components including misfolded proteins and damaged organelles, to maintain the intracellular homeostasis and normal cellular function. Autophagy plays a crucial role in maintaining follicular growth, oocyte development, and the reproductive cycle in the ovary. Autophagy regulates follicular development by various classical pathways, such as PI3K/AKT/mTOR, AMPK, MAPK/ERK1/2. Among these mechanisms, PI3K/AKT/ mTOR is considered as the most important mechanism to regulate autophagy during follicle development. Inhibition of the PI3K/AKT/mTOR pathway stimulates the occurrence of autophagy accompanied by apoptosis results in the degeneration of GCs (granulosa cells), which subsequently trigger follicular atresia [18, 19]. Additionally, abnormal expression of autophagy key regulator induces the perturbation of primordial follicular pool, a decline of oocyte maturation competence and capacity of successful fertilization. In mice with a deficiency in *Atg7*, a key protein involved in autophagy, the ovaries showed a decreased level of autophagy, resulting in a significant reduction in oocyte and germ cells, which accompanied by producing small litters or infertility [20]. Studies have reported that Zn not only involved in autophagy, but is also important for autophagy induction. Imbalance of Zn may be accompanied by its accumulation in the lysosomes, which then lead to the disruption of lysosomal membranes and the release of lysosomal hydrolases into cytosol, eventually triggering apoptosis [21, 22]. In H9c2 cell, Zn has been shown to involve in the activation of mitophagy through regulating PINK1 and BECLIN 1, thus preventing the generation of excess superoxide in mitochondria [23]. In hypoxia-induced myocytes, Zn reduced the generation of superoxide through inducing mitochondrial autophagy in mitochondria, thus protecting the cells from oxidative stress damage [24].

In the present study, the Zn-deficiency mice model was built by using Zn-deficient diet to explore the potential regulating mechanism of Zn in ovarian development and function. We found that Zn deficiency caused the aberrant hormone secretion and follicle maturation by disrupting mitochondrial function and autophagy. More importantly, our findings demonstrated that Zn deficiency-caused ovarian dysfunction could be reversed by administration of zinc glycine.

## Materials and methods

### Animals and diets

Eighty female ICR mice, three-week-old, were purchased from Hangzhou Medical College, China. The mice were handled and utilized in accordance with the guidelines of Institutional Animal Care and Use Committee at Zhejiang A&F University, China. The mice were randomly divided into three groups: AIN-93G zinc-control diet (38 ppm, ZnC), zinc-deficient diet (1ppm, ZnD), and a zinc-salvage group (ZnS) receiving zinc glycine (Aladdin, 14281-83-5) supplementation through gavage at a dose of 25 mg/kg. All groups had free access to deionized water. After a period of 30 days, the mice were used for subsequent experiments.

### Real-time quantitative PCR (RT-qPCR)

Total RNA was extracted from the ovaries using the MolPure® Cell/Tissue Total RNA Kit (19221ES50) following the manufacturer’s instructions. The first-strand cDNA was generated using cDNA Synthesis SuperMix (YEASEN). Relative quantitative analysis was performed using 2X Universal SYBR Green Fast qPCR Mix (Abclonal, China, RK21203) on a QuantStudio 5 instrument (Applied Biosystems, Carlsbad, CA, USA). The PCR cycling parameters were as follows: an initial denaturation at 95 °C for 10 min, followed by 40 cycles of denaturation at 95 °C for 15 s, annealing and extension at 60 °C for 60 s, and a final melting curve analysis with denaturation at 95 °C for 15 s and annealing at 60 °C for 1 min; *Gapdh* was used to normalize gene expression levels. The relative transcript abundance was analyzed via the 2^−ΔΔCT^ method. Primers used in this study are shown in Table S1.

### Immunoblotting

Proteins were extracted from ovaries with RIPA Lysis Buffer (Beyotime, P0013C), and protein concentration was adjusted by BCA quantification (Beyotime, P0010S) and denatured at 95 °C for 10 min. Samples were separated on 10% polyacrylamide gels (20325ES62, YEASEN) and blotted on PVDF membranes (Immobilon-P, Millipore). Incubate in 5% skim milk for 1 h, then incubate with primary antibody overnight at 4 °C. Then TBST was washed 3 times for 5 min each, and the secondary antibody was incubated at room temperature for 2 h and washed again with TBST. The HyperSignal ECL chemiluminescence kit (4 A Biotech Co., Beijing.) was used to develop the color and the chemiluminescence signals were captured with a Tanon 4600 chemiluminescence imaging system (Tanon, Beijing, China). The Primary antibodies used in this study are presented in Table S2.

### Hematoxylin and eosin staining

The ovaries were fixed in 4% paraformaldehyde in PBS for more than 24 h at room temperature. After dehydration in graded ethanol and clearing in xylene, the ovaries were embedded in paraffin; Subsequently, the samples were sliced on 3-µm thickness using a microtome, and then stained with hematoxylin and eosin (H&E). The stained sections were examined under an optical microscope (Olympus, IX73). Follicle counting estimated the number of follicles at various stage within each ovary by double-blind assessment the number of follicles at each level in the H&E-stained images of complete ovarian sections. The criteria used to classify the follicles: Primordial follicles consist of a singular layer of squamous granulosa cells, pre-antral follicles consist of cuboidal granulosa cells layer and a thecal layer, antral follicles consist of an oocyte surrounded by a fluid-filled antrum and layers of granulosa cells, and atretic follicles exhibit dissolved oocyte cytoplasm with collapsed follicle walls.

### ELISA

The serum level of Anti-Müllerian hormone (AMH) was determined using the AMH ELISA Kit (Mlelisa, YJ037597, China). a total of 10 µL of mouse serum from the control and ZnD treated group were taken, and the assay was performed following the manufacture’s protocol. Briefly, the standard was diluted, and the microelisa strip plate was set up with blank wells, standard wells and sample wells. After adding the standard and sample, the plate was incubated at 37℃ for 30 min. The plate was then washed 5 times with the wash solution and 50 µL enzyme-labeled reagent was added to each well, followed by incubation at 37℃ for 30 min. After washing the plate 5 times, 50 µL chromogen solution A and B were added to each well, and the plate was incubated at 37℃ for 10 min. Lastly 50 µL stop solution was added to each well, and the absorbance of each well was measured at 450 nm. A standard curve was drawn, and the AMH concentration of the tested samples was calculated.

### Immunofluorescence (IF) staining

The dehydrated sections used for histopathology evaluation and the permeabilized cells were subjected to IF staining to detect apoptosis. In brief, the sections were subjected to antigen retrieval in EDTA using microwave treatment, and autofluorescence was quenched with AutoFluo Quencher. Subsequently, the sections and cells were blocked with BSA. The samples were then incubated overnight with a primary antibody against γH2AX (WL00616a; 1:200) at 4℃. After that, a secondary antibody (Alexa Fluor® 488) was added and incubated in the dark for 50 min at room temperature. DAPI was used to counterstain the samples, which were then observed under confocal microscope (FV3000, Olympus).

### Mouse oocytes collection and culture

Mice were treated and handled in accordance with the guidelines of Institutional Animal Care and Use Committee at Zhejiang A&F University, China. Female ICR mice were intraperitoneally injected with 5 IU of pregnant mare serum gonadotropin (PMSG). After 48 h, germinal vesicle (GV) cumulus-oocyte complexes (COCs) were quickly isolated from ovaries in M2 medium containing 3-Isobutyl-1-methylxanthine (IBMX). The cumulus cells were then removed from COCs by repeated mouth-controlled pipetting. The oocytes were cultured in IBMX-free M16 medium (Sigma-Aldrich) under mineral oil at 37 ℃ in 5% CO2 atmosphere.

### Detection of intracellular Zn^2+^ and ROS

Zinc ions concentration and ROS levels were detected using Zinpyr-1 (ChemCruz, sc-213,182) and Reactive Oxygen Detection Kit (Beyotime, S0033M-1). Zinpyr-1 (10 µM) and ROS probe (10 µM) were incubate with samples for 30 min at 37 °C, followed by three washes with M 2 medium. The fluorescent signals were detected using a confocal microscope.

### Statistical analysis

The statistical analysis was performed with GraphPad Prism software 9 (La Jolla, USA). Comparisons of data was performed by one-way analysis of variance (ANOVA) with Tukey’s multiple comparison test or two tailed unpaired t-test, *p* values < 0.05 was considered significant. Data are presented as the mean ± standard deviation (SD) of the triplicate experiments.

## Results

### Zinc deficiency affected ovarian follicles development and hormones secretion

**Fig. 1:**
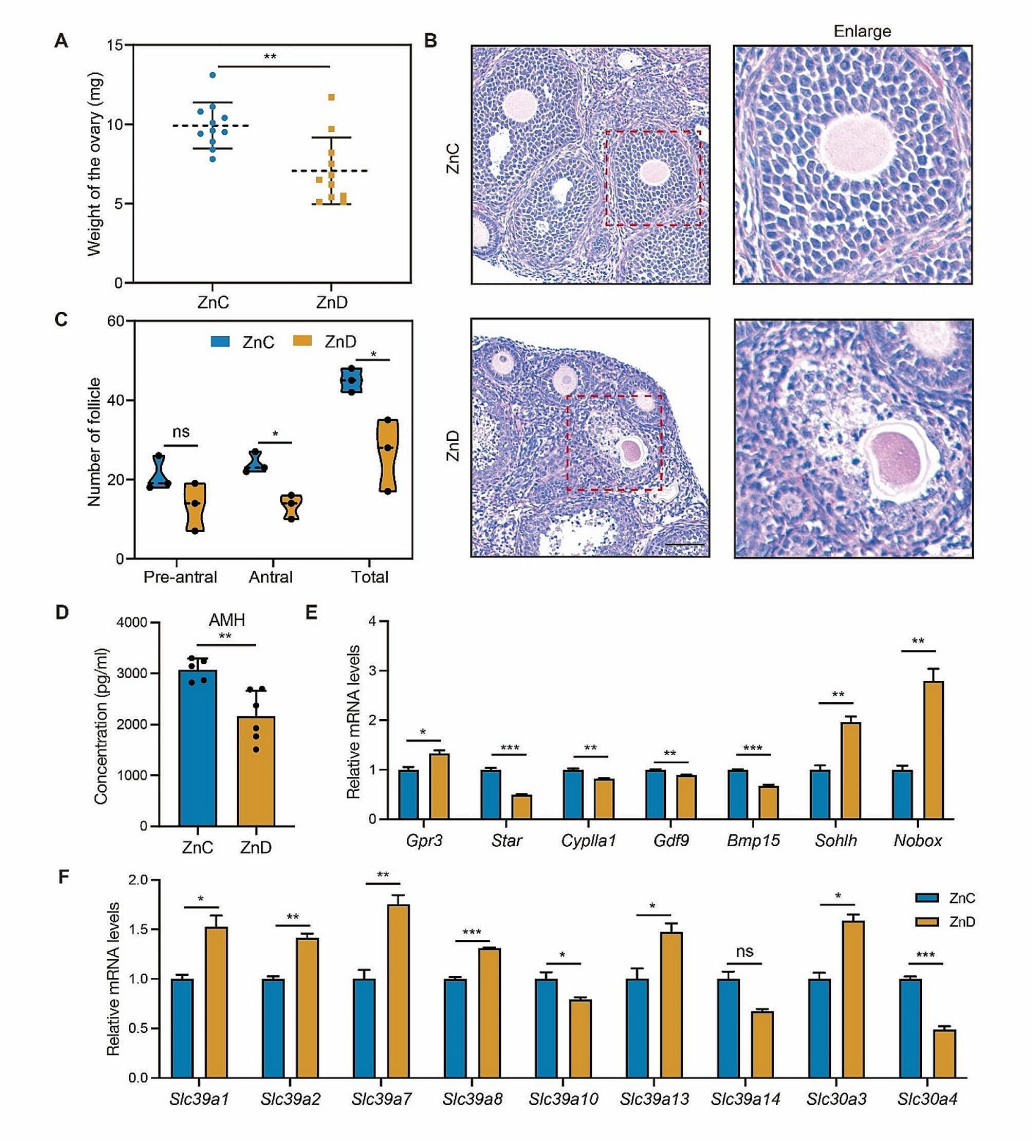
Zinc deficiency impaired ovarian follicles maturation and hormones secretion. **A** Ovarian weight in ZnC and ZnD groups. **B** Representative H&E histochemical section images of ovaries from ZnC and ZnD groups. Bar = 120 μm. **C** Quantification of the number of pre-antral and antral follicles in ZnC and ZnD groups. **D** Quantification of AMH concentration in ZnC and ZnD groups. **E** mRNA levels of *Gpr3, Star, Cyp11a1, Gdf9, Bmp15, Sohlh* and *Nobox* were evaluated by RT-PCR in ZnC and ZnD groups. **F** mRNA levels of Zinc transporters were evaluated by RT-PCR in ZnC and ZnD groups. *p < 0.05, **p < 0.01, ***p < 0.001. Data are represented as mean ± SD from at least three independent experiments. ZnC: control; ZnD: zinc deficiency

To investigate the impact of zinc deficiency on ovarian function, mice were subjected to a marginal zinc deficiency diet for 4 weeks. As shown in Fig. 1A, the ovarian weight was significantly reduced in the mice with zinc deficiency. Furthermore, we conducted hematoxylin and eosin staining of ovarian sections to assess ovarian development. As shown in Fig. 1B-C, the control group ovaries display normal morphology of follicles at different developmental stages, whereas zinc deficiency disrupted follicular development, leading to a decreased number of pre-antral and antral follicles. The reduced concentration of anti-Müllerian hormone (AMH) in the serum further supported that the ovarian reserve was diminished in zinc deficiency mice (Fig. 1D). Additionally, we examined the gene expression levels related to ovarian hormone secretion. Through RT-qPCR analysis, we observed a significant increase in the mRNA level of *Gpr3*, while the expression levels of *Star* and *Cyplla1* were markedly reduced in zinc deficiency mice (Fig. 1E). Consistently, zinc deficiency also affected the expression levels of genes involved in ovarian development. As shown in Fig. 1E, the mRNA levels of *Gdf9* and *Bmp15* were down-regulated in zinc deficiency mice, whereas the expression levels of *Sohlh* and *Nobox* were remarkably up-regulated. Interestingly, we also observed the change in the mRNA levels of zinc transporters in the ovaries from zinc-deficient mice. As shown in Fig. 1F, zinc deficiency significantly reduced the mRNA levels of *Slc39a1*, *Slc39a2*, *Slc39a7*, *Slc39a8*, *Slc39a13* and *Slc30a3.* Conversely, the expression of *Slc39a10* and *Slc39a14* and *Slc30a4* was found to be increased, suggesting a disruption in zinc transport in the ovaries of zinc-deficient mice. Collectively, these results highlight the critical role of zinc in ovarian follicular development and function in mice.

### Zinc deficiency disrupted redox homeostasis in the ovary of mice

**Fig. 2:**
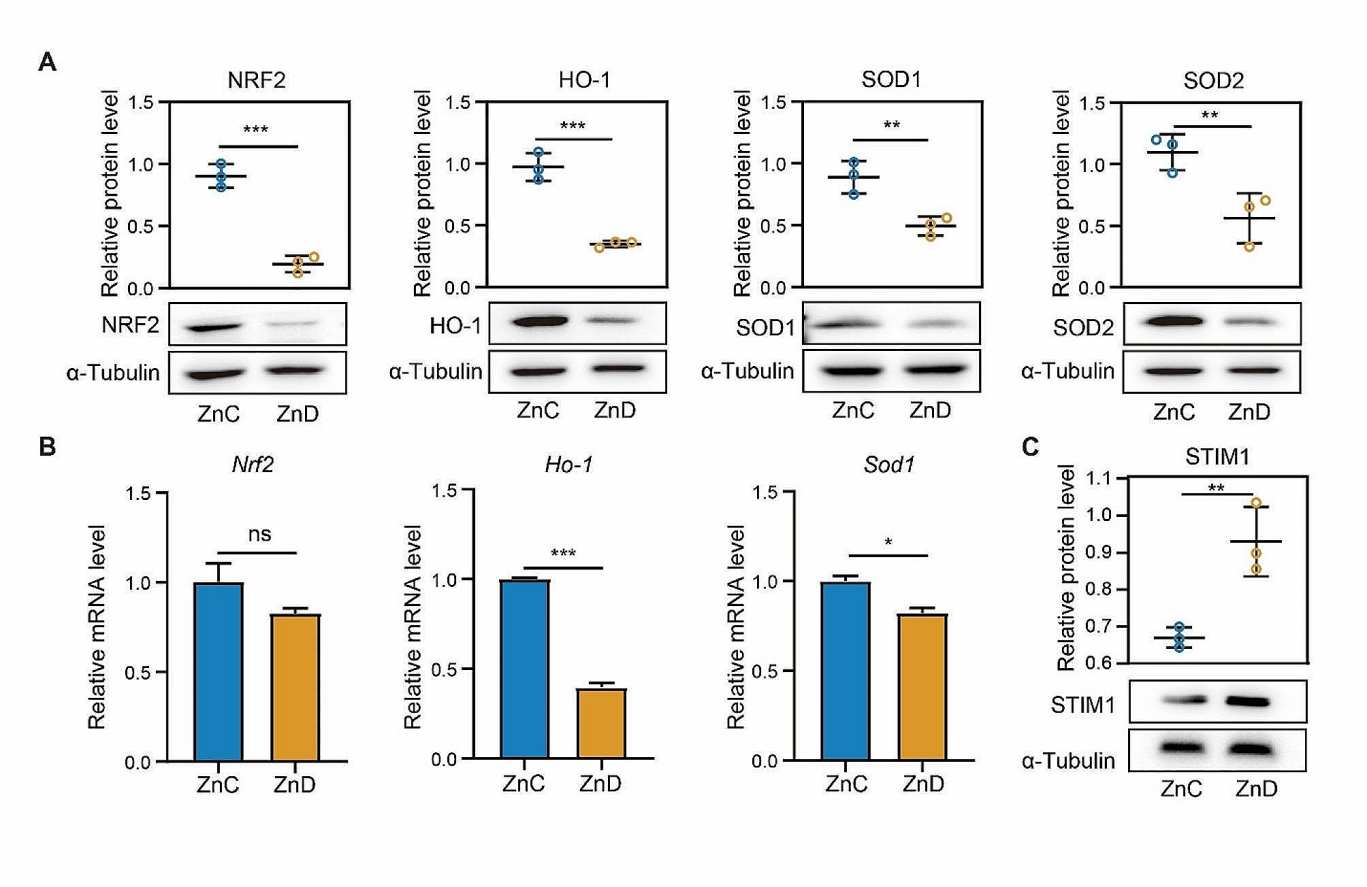
Zinc deficiency disrupted redox homeostasis in the ovary of mice. **A** Protein levels of NRF2, HO-1, SOD1 and SOD2 were determined by western blot in ZnC and ZnD groups. **B** mRNA levels of *Nrf2*, *Ho-1* and *Sod1* were evaluated by RT-PCR in ZnC and ZnD groups. **C** STIM1 protein level was determined by western blot in ZnC and ZnD groups. *p < 0.05, **p < 0.01, ***p < 0.001. Data are represented as mean ± SD from at least three independent experiments. ZnC: control; ZnD: zinc deficiency

Given that oxidative stress is major causal factors in ovarian dysfunction, we conducted immunoblotting to determine the expression of nuclear factor E2-related factor 2 (NRF2), a key transcription factor involved in the cellular response to oxidative stress. Our findings revealed a significant reduction in the protein level of Nrf2 in the ovaries of zinc-deficient mice. NRF2 plays a critical roles in activating antioxidant enzymes, such as Heme oxygenase-1(HO-1) and superoxide dismutase (SOD), which help maintain redox homeostasis. Therefore, we compared the protein levels of HO-1 and SOD1/2 in the control and zinc deficiency groups. Consistently, our results showed that zinc deficiency greatly reduced the protein expression of HO-1 and SOD1/2 in the ovaries (Fig. 2A). We also used RT-PCR was to assess the mRNA levels of these genes. Interestingly, we observed a significant reduction in the mRNA levels of *Ho-1* and *Sod1* in the zinc deficiency group, while there were no changes in the *Nrf2* expression (Fig. 2B). This implied that zinc deficiency affected the protein level of NRF2-HO1/SOD1/2 signaling pathway. Furthermore, we examined the protein expression level of STIM1, which acted as a calcium store sensor and mediates cellular responses to reactive oxygen species (ROS). We found a significantly increase in STIM1 expression due to zinc deficiency (Fig. 2C). Taken together, our observations indicated that zinc deficiency induces oxidative stress in the ovaries by inhibiting the NRF2-HO1/SOD1/2 signaling pathway.

### Zinc deficiency induced abnormal mitochondrial dynamics and mitophagy in the ovary of mice

**Fig. 3:**
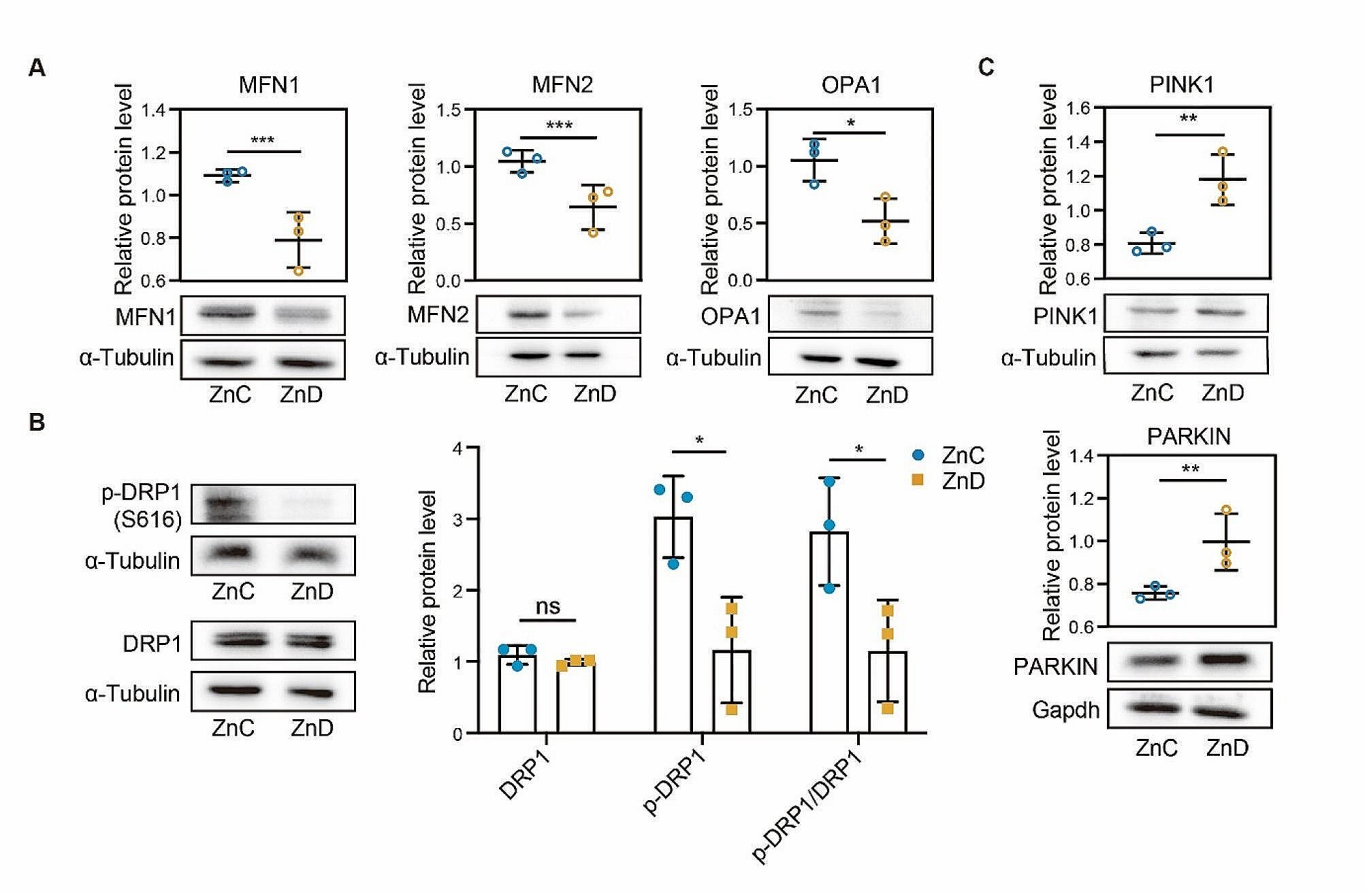
Zinc deficiency induced abnormal mitochondrial dynamics and mitophagy in the ovary of mice. **A** Protein levels of MFN1, MFN2 and OPA1 were determined by western blot in ZnC and ZnD groups. **B** Protein levels of pDRP1(616) and DRP1 were determined by western blot in ZnC and ZnD groups. **C** Western blot was used to determine the protein levels of PINK1 and PARKIN. *p < 0.05, **p < 0.01, ***p < 0.001. Data are represented as mean ± SD from at least three independent experiments. ZnC: control; ZnD: zinc deficiency

The continuous process of mitochondrial fission and fusion plays a crucial role in maintaining a balance between mitochondrial biogenesis and apoptosis, thereby contributing to mitochondrial dynamics. To explore the potential mechanism of oxidative stress induced by dietary zinc deficiency, we accessed the levels of protein involved in mitochondrial fusion and fission process. As shown in Fig. 3A, we found that mice with zinc deficiency exhibited significantly reduced protein levels of mitofusin1/2 (MFN1/2) and optic atrophy 1 (OPA1) in the ovaries, suggesting impaired mitochondrial dynamics. Additionally, we observed a marked reduction in the phosphorylation of mitochondrial fission-related protein DRP1 in the ovaries of zinc-deficient mice (Fig. 3B). Furthermore, we explored the occurrence of mitophagy, a specific form of autophagy targeting mitochondria, in response to zinc deficiency in the ovaries. Our findings demonstrated a significant increase in the levels of PINK1 and PARKIN proteins in the ovaries of mice with zinc deficiency, indicative of mitophagy induction (Fig. 3C). Based on these observations, we proposed that zinc deficiency disrupted ovarian function by disturbing mitochondrial dynamics and further inducing mitophagy in the ovaries.

### Zinc deficiency inhibited the occurrence of ovarian autophagy in mice

**Fig. 4:**
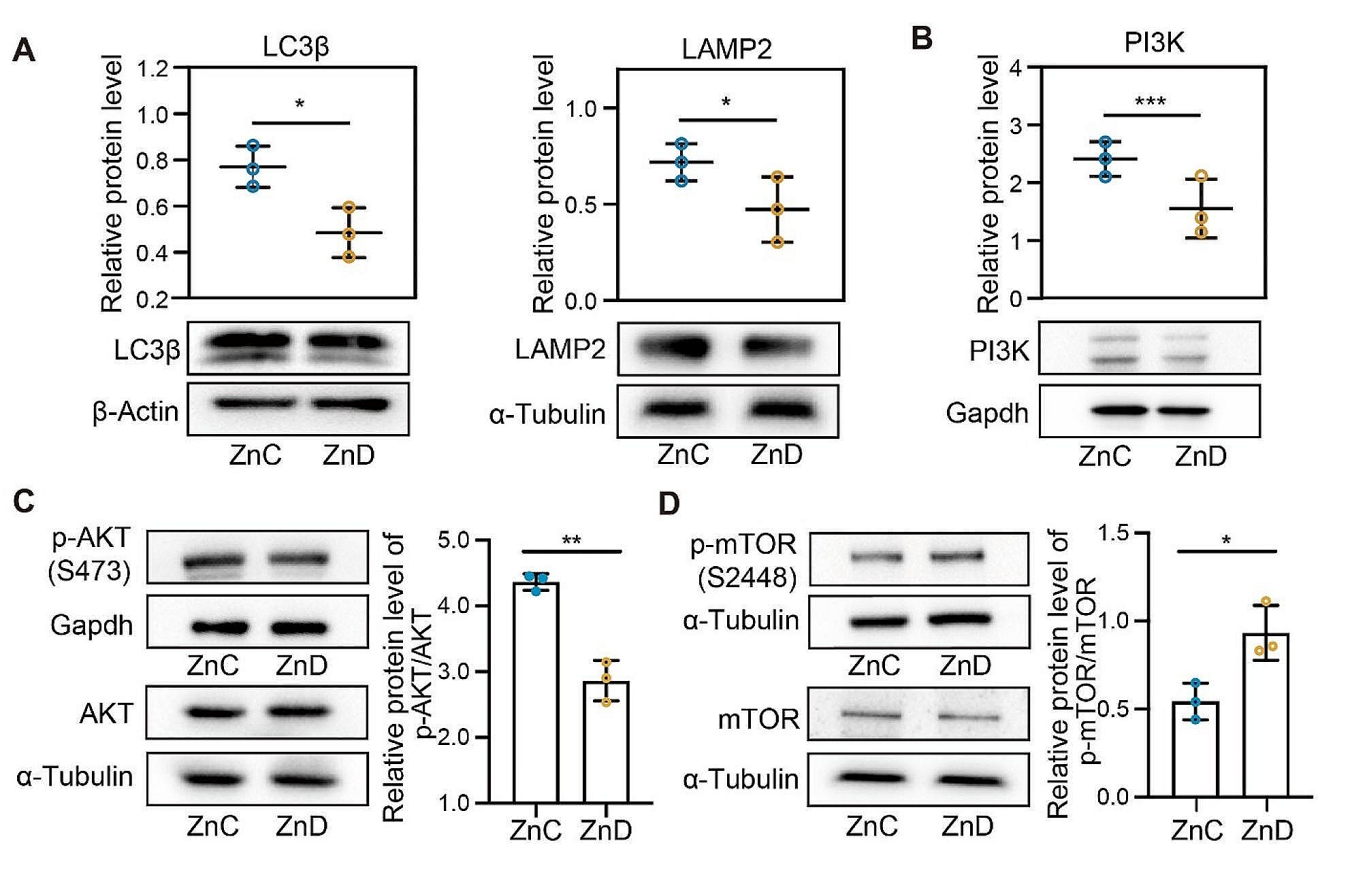
Zinc deficiency inhibited the occurrence of ovarian autophagy in mice. **A** Protein levels of LC3(β)and LAMP2 were examined by western blot in ZnC and ZnD groups. **B-D** PI3K, p-AKT(S473)and AKT, p-mTOR (S2448) and mTOR protein levels were examined by western blot in ZnC and ZnD groups. *p < 0.05, **p < 0.01, ***p < 0.001. Data are represented as mean ± SD from at least three independent experiments. ZnC: control; ZnD: zinc deficiency

Given the evidence that aberrant mitochondrial function often led to defects in autophagosome-lysosome fusion, we sought to investigate the impact of zinc deficiency on the ovarian autophagy. We focused on LC3β, which serves as a direct indicator of cellular autophagy since it is the only protein retained on the bilayer membrane of autophagosomes during their formation and progression. As shown in Fig. 4A, we found that the protein level of LC3β was significantly reduced in the ovaries of mice with zinc deficiency. Furthermore, we noted a significant decrease in the expression level of lysosome-associated membrane protein 2 (LAMP 2), which suggested that zinc deficiency inhibited the formation of autophagosome in ovary.

Since PI3K/AKT/mTOR plays a vital role in autophagy regulation during follicle development, we examine the impact of zinc deficiency on this pathway. As shown in Fig. 4B-C, our data showed that protein levels of PI3K and phosphorylated AKT were significantly reduced in the ovaries of zinc-deficient mice, along with increased levels of mTOR phosphorylation (Fig. 4D). Thus, our data indicated that zinc deficiency inhibits autophagy by activating the PI3K/AKT/mTOR signaling pathway.

### Dietary zinc deficiency led to increased DNA damage and apoptosis in the ovary of mice

**Fig. 5:**
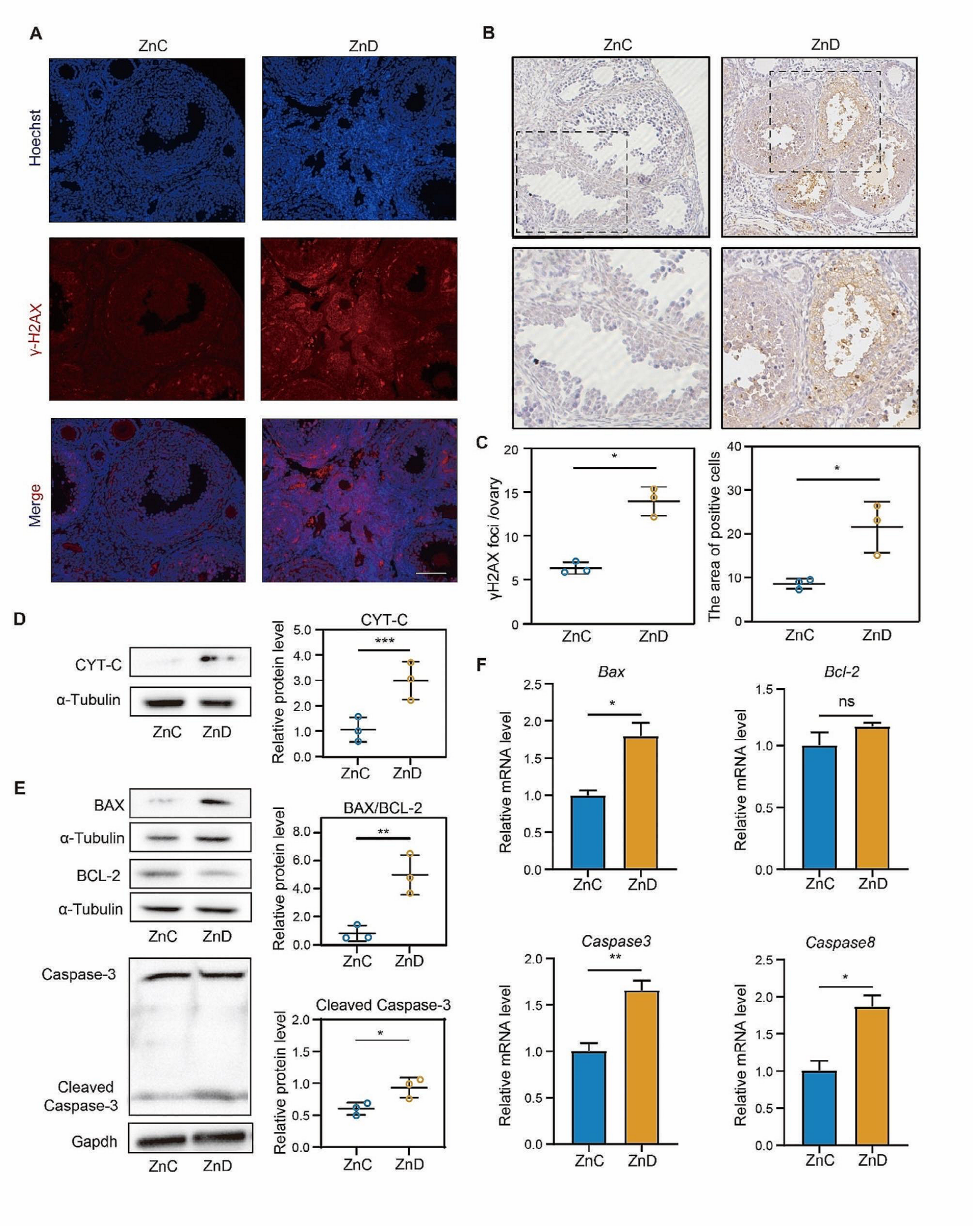
Dietary zinc deficiency led to increased DNA damage and apoptosis in the ovary of mice. **A** Representative images of γH2AX staining of ovarian tissue from ZnC and ZnD groups. Scale bar, 80 μm. **B** Representative images of TUNEL staining of ovarian tissue from ZnC and ZnD groups. Scale bar, 80 μm. **C** Number of γH2AX foci and the percentage of TUNEL positive regions were quantified in ZnC and ZnD groups. **D-E** Protein levels of CYT-C, BAX and BCL-2, Cleaved CASPASE-3 and CASPASE-3 protein levels were examined by western blot in ZnC and ZnD groups. **F** mRNA levels of *Bax, Bcl-2, Caspase 3* and *Caspase 8* were evaluated by RT-PCR in ZnC and ZnD groups. *p < 0.05, **p < 0.01, ***p < 0.001. Data are represented as mean ± SD from at least three independent experiments. ZnC: control; ZnD: zinc deficiency

To investigate the effect of zinc efficiency on apoptosis, we first explored the occurrence of DNA damage in the ovaries by staining γH2AX. Our results revealed a significantly increase in γH2AX foci in the ovarian section with zinc deficiency, indicating the presence of DNA damage (Fig. 5A). Furthermore, immunohistochemistry results showed a significant increase in TUNEL-positive area in the ovarian of zinc- deficient mice, suggesting an increase in apoptotic cells (Fig. 5B). One of key proteins involved in apoptosis is cytochrome c, which is released from the mitochondria into the cytosol, triggering programmed cell death. We therefore examined the protein levels of cytochrome c in the ovaries with zinc deficiency and found a significantly increase compared to the control group (Fig. 5D). This finding further supported the occurrence of apoptosis in the ovarian of zinc-deficient mice. Additionally, our immunoblotting results demonstrated that the protein levels of BAX/BCL2 and Cleaved CASPASE-3 and CASPASE-3 was significantly up-regulated in ovary of zinc-deficient mice (Fig. 5E). Consistently, RT-qPCR results showed that zinc deficiency significantly increased the mRNA levels of *Bax*, *Caspase-3*, and *Caspase-8* in ovaries (Fig. 5F). Taken together, our data indicated that zinc deficiency led to ovarian dysfunction by inducing apoptosis.

### Supplementation of zinc glycine restored the in vitro maturation competency of oocyte from zinc-deficient mice

**Fig. 6:**
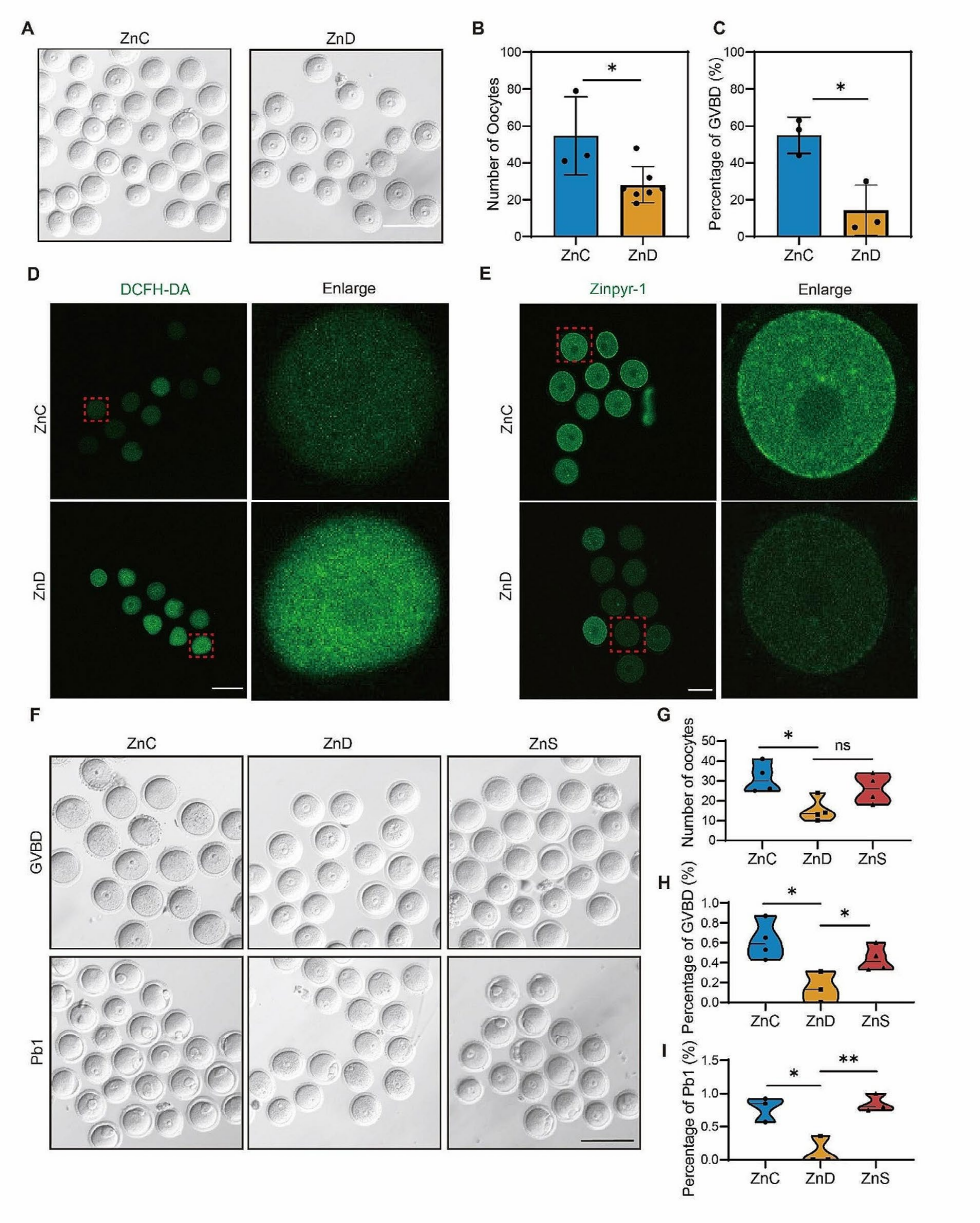
Supplementation of zinc glycine restores the in vitro maturation competency of oocyte from zinc-deficient mice. **A** Representative DIC images of germinal vesicle breakdown in ZnC and ZnD groups. Scale bar, 110 μm. **B-C** Quantification of oocyte number and rate of GVBD in ZnC and ZnD groups. **D** Representative images of DCFH-DA staining in ZnC and ZnD groups. Scale bar, 80 μm. **E** Representative images of cytosolic Zn^2+^ levels in ZnC and ZnD groups. Scale bar, 80 μm. **F** Representative DIC images of GVBD and first polar body extrusion after ZnS supplementation. Scale bar, 110 μm. **G** Quantification of oocyte number after ZnS supplementation. **H-I** Percentage of GVBD and polar body extrusion ZnS supplementation. Scale bar, 80 μm. *p < 0.05, **p < 0.01, ***p < 0.001. Data are represented as mean ± SD from at least three independent experiments. ZnC: control; ZnD: zinc deficiency

As the ovarian development was inhibited in zinc-deficient mice, we then assessed its effect on oocyte quality. As shown in Fig. 6A-C, we found a dramatic reduction in oocyte number, accompanied by a decrease in the percentage of germinal vesicle breakdown (GVBD) in these mice. Additionally, we used DCFH-DA dye to measure the level of reactive oxygen species (ROS) in the oocyte and found that the ROS levels was remarkably increased in the oocyte from zinc-deficient mice compared to the control group (Fig. 6D). Furthermore, a notable decrease in the concentration of zinc ions was observed in the oocytes of zinc-deficient mice (Fig. 6E). These findings indicated the crucial role of zinc ions in maintaining redox homeostasis and regulating oocyte maturation. In contrast, supplementation of zinc glycine significantly restored GVBD and first polar body (PB1) extrusion in oocytes from zinc-deficient mice (Fig. 6F-I), suggesting that zinc glycine supplementation can effectively rejuvenate the quality of oocytes in zinc-deficient conditions.

## Discussion

The development of ovarian follicles is a complex process that has been influenced by various factors to produce high quality oocytes, ensuring successful fertilization and implantation [25]. This process involves the growth and maturation of ovarian follicles. Previous studies have reported the concentration of zinc iron was low in the serum of patients with polycystic ovarian syndrome (PCOS), which is accompanied by abnormal antral follicle development, low oocyte quality and infertility [26]. However, the impact of changes in zinc ions concentration on ovarian development is still largely unknown. To address this, we established a mice model of marginal zinc deficiency to examine its effect on ovary development. Our findings revealed that the follicle maturation was inhibited in the ovaries of zinc-deficient mice, which was accompanied by the abnormal oocyte resumption. Dietary of ZnS could rescue zinc deficiency-induced aberrant oocyte maturation.

The activation of the pSMAD2/3 signaling pathway in granulosa cells, which is crucial for cumulus cell proliferation and expansion, was disrupted due to zinc deficiency [27]. Hence, we hypothesized that abnormal proliferation of cumulus and granulosa cells contributed to the impaired follicle development observed in zinc-deficient mice. Interestingly, we also observed diminished levels of anti-Müllerian hormone (AMH), as well as reduced expression of genes related to steroid and cholesterol synthesis in the ovaries of zinc-deficient mice. This indicated that zinc deficiency also affected hormone synthesis, which was consistent with previous research showing zinc was involved in progesterone secretion from granulosa cells and the production of follicle-stimulating hormone (FSH), both of which regulated follicular growth and maturation [28, 29]. Oxidative stress is a major contributor to granulosa cell dysfunction, apoptosis, follicular atresia, and reduces follicle count in each developmental stage [30]. Zinc has been shown to enhance the activation of antioxidant enzymes, such as catalase (CAT) and superoxide dismutase (SOD), while reducing the activities of oxidant-promoting enzymes like inducible nitric oxide synthase (iNOS) and nicotinamide adenine dinucleotide phosphate (NADPH) oxidase [31, 32]. Nuclear factor erythroid 2-related factor 2 (NRF2) plays a crucial role in regulating the antioxidant response. Meanwhile, the NRF2-HO1 signaling pathway is inhibited in the granulosa cells of polycystic ovary syndrome (PCOS) [33]. Our findings showed that zinc deficiency can inhibit the NRF2-HO1/SOD1/2 signaling pathway, indicating that zinc acted as an essential anti-oxidative element in the ovaries, maintaining the redox homeostasis.

Mitochondria are highly dynamic organelles that constantly undergo fusion and fission to maintain homeostasis in response to changes in the cellular environment. The fusion of mitochondria is orchestrated by proteins such as MFN1, MFN2, and OPA1, while fission is regulated by a protein called DRP1 [34]. Mitochondrial fission plays a crucial role in isolating damaged mitochondria, protecting the healthy portion of mitochondria by inhibiting the spread of depolarization and reactive oxygen species (ROS) [35]. In contrast, mitochondrial fusion acts as a restorative mechanism to restore a part of mitochondria from reversible damage. Inhibition of mitochondrial fission by depletion of Drp1 caused the failure of follicular maturation and aberrant mitochondria function in oocyte [36–38]. Additionally, MFN1/2 was also demonstrated to be required for maintaining ovarian follicular reserve and oocyte growth [39, 40]. Our findings showed that zinc deficiency both inhibited the mitochondrial fission and fusion process by reducing the protein levels of MFN1/2 and the phosphorylation of DRP1 in ovaries, suggesting that zinc was crucial for maintaining mitochondrial quality during the follicular development.

Once the damaged mitochondria were segregated by mitochondrial fission, it will be removed by autophagy-mediated lysosomal degradation. Impaired autophagy caused the accumulation of damaged mitochondria in cell which further led to the occurrence of apoptosis. PI3K/AKT/mTOR pathway is a highly conserved pathway, that plays a crucial role in modulating cell growth, apoptosis, autophagy and metabolism in ovarian granulosa cells (GCs). It has been shown that the PI3K-AKT pathway can inhibit autophagy by activating mTOR, a major negative regulator of autophagy [41]. However, it is unclear whether zinc deficiency can activate the PI3K-AKT pathway to inhibit autophagy in ovaries. In the present study, we found that zinc deficiency inhibited autophagy in ovaries by activating PI3K/AKT pathway. This activation was accompanied by increased phosphorylation of mTOR, suggesting that inhibiting the PI3K/AKT/mTOR axis may be a critical way to rescue zinc deficiency-induced inhibition of autophagy. Previous studies have shown that inhibiting mTOR enhances autophagy and suppress apoptosis [42, 43]. Conversely, abnormal autophagy impairs mitochondrial function and leads to apoptosis. Consistently, our result showed that zinc deficiency induced DNA damage and apoptosis by inducing mitochondria-related apoptosis. Previous studies have reported that changes in zinc transport proteins were associated with the occurrence of autophagy [44]. Knockdown of Zip13 in fibroblasts reduced the expression of LC3-I/II and caused severe autophagy defects [45]. Moreover, inhibition of Zip1 induced mitophagy by reducing mitochondrial membrane potential, suggesting that Zinc transport proteins play crucial roles in regulating autophagy [46]. In our study, we also observed changes in the mRNA level of several zinc transporter-related genes, such as *Slc39a1*, *Slc39a7, Slc30a3*, suggesting a correlation between changes in zinc transport genes and autophagy in zinc-deficient ovaries. However, the potential regulatory mechanism needs to be further explored in future research.

## Conclusions

In summary, this study highlights the critical role of zinc as a trace element in maintaining ovarian function. Zinc deficiency was found to disrupt follicle maturation and mitochondrial function, which further inhibited autophagy. Importantly, zinc deficiency was also associated with the failure of oocyte maturation, which was rescued by supplementation with ZnS. These findings significantly contribute to our understanding of the impact of zinc deficiency on female reproductive capacity in mammals.

### Electronic supplementary material

Supplementary table 1

Supplementary table 2

## Data Availability

No datasets were generated or analysed during the current study.
