# Supplementary material for: Zinc deficiency deteriorates ovarian follicle development and function by inhibiting mitochondrial function
Source: J Ovarian Res. 2024 May 28;17:115. doi: 10.1186/s13048-024-01442-z (PMC11134637; doi:10.1186/s13048-024-01442-z)
Supplement: Supplementary file 1 — Supplementary table 1 [file 13048_2024_1442_MOESM1_ESM.docx]

| Gene name | F：5′-3′ | R：5′-3′ |
| --- | --- | --- |
| Gpr3 | CCTGCTGGTGGGTAGCTTG | CATCACATAAGTCCGAGTTACCG |
| Star | CGCAGAGGTTCCACCTGTGT | TCCGGCATCTCCCCAAA |
| Cyplla1 | AGGTCCTTCAATGAGATCCCTT | TCCCTGTAAATGGGGCCATAC |
| Gdf9 | TCTTAGTAGCCTTAGCTCTCAGG | TGTCAGTCCCATCTACAGGCA |
| Bmp15 | TCCTTGCTGACGACCCTACAT | TACCTCAGGGGATAGCCTTGG |
| Sohlh | TCTCAGCCACATCACAGAGG | GGGGACGCGAGTCTTATACA |
| Hobox | CTATCCTGACAGTGACAAACGCC | CACCCTCTCAGCACCCTCATTAT |
| Nrf2 | CAGCATAGAGCAGGACATGGAG | GAACAGCGGTAGTATCAGCCAG |
| Ho-1 | CACTCTGGAGATGACACCTGAG | GTGTTCCTCTGTCAGCATCACC |
| Sod1 | ATGGCGATGAAAGCGGTG | CCTTGTGTATTGTCCCCATACTG |
| Opa1 | ATACTGGGATCTGCTGTTGG | AAGTCAGGCACAATCCACTT |
| Drp1 | TTACGGTTCCCTAAACTTCACG | GTCACGGGCAACCTTTTACGA |
| Bax | CCAGGACGCATCCACCAAGAAG | GCTGCCACACGGAAGAAGACC |
| Bcl-2 | AGGGGCTACGAGTGGGATACT | GACGGTAGCGACGAGAGAAG |
| Caspase3 | TTGAGAACACGGAGAGTG | CACAGTCAAGGCTTGTCCAGA |
| Caspase8 | CTGGCATTACGCCGTGATC | CATCAGTGAAGAGTCTGCGAG |
| Slc39a1 | GACGTGGTCAGGGACATTAG | AAAGGTGAGGACAGGAGAGG |
| Slc39a2 | AGCTACAGGTCATCACCACAGG | TGCATCAACCCTGCTCCCAA |
| Slc39a7 | GAAGCTCCATCTTTGCCTTCTG | TTAGGTGGGAGCAGTGTTAAGG |
| Slc39a8 | AGCTTGAACACACCCTGCAGAA | AATCCCAGAGCATGGCAAGAC |
| Slc39a10 | GCATCAGCACATCCATAGCC | TTGGCATACTGACCGACTGC |
| Slc39a13 | AGGCCCCCAGCAAAGACCCCA | CTTTTTGCTCACAAGGAAGCT |
| Slc39a14 | TGGTGCCTCCTTCACTGTGT | AGGCCAGACCCAAATAGCAG |
| Slc30a3 | CCGCCTCCACGTACCGCTCCACCTGCACATG | GGCCGTCAGCCTCTTCTCGCTCTGGGTGTC |
| Slc30a4 | GCTGCCGTCCTCTACTTG | GCATGACATCGCCGTTTA |
| GAPDH | CCCCAATGTGTCCGTCGTG | TGCCTGCTTCACCACCTTCT |

Table S1. Primer sequences for RT-qPCR
