# Supplementary material for: Zinc deficiency deteriorates ovarian follicle development and function by inhibiting mitochondrial function
Source: J Ovarian Res. 2024 May 28;17:115. doi: 10.1186/s13048-024-01442-z (PMC11134637; doi:10.1186/s13048-024-01442-z)
Supplement: Supplementary file 2 — Supplementary table 2 [file 13048_2024_1442_MOESM2_ESM.docx]

| Protein | Company | Catalogue Number | Dilution |
| --- | --- | --- | --- |
| Nrf2 | Huabio | ER1706 | 1:1000 |
| Ho-1 | Abclonal | A1346 | 1:1000 |
| Sod1 | Wanleibio | WL01846 | 1:1000 |
| Sod2 | Proteintech | 24127-1-Ig | 1:5000 |
| Stim1 | Huabio | ET1612-53 | 1:1000 |
| Mfn1 | Proteintech | 66776-1-Ig | 1:2000 |
| Mfn2 | Huabio | ER1802-23 | 1:2000 |
| Opa1 | Huabio | ET1705-9 | 1:1000 |
| Cyt-c | Wanleibio | WL02229 | 1:500 |
| p-Drp1(616) | Cell Signaling | 3455S | 1:1000 |
| Drp1 | Cell Signaling | 8570S | 1:2000 |
| Lc3(β) | Abclonal | A19665 | 1:1000 |
| Pink1 | Abclonal | A7131 | 1:1000 |
| Parkin | Santa | SC-32282 | 1:500 |
| Pi3k | Beyotime | AF7742 | 1:1000 |
| p-AKT(S473) | Wanleibio | WLP001a | 1:1000 |
| Lamp2 | Santa | sc-19991 | 1:500 |
| Akt | Zenbio | 382804 | 1:1000 |
| p-mTOR（S2448） | Abcam | ab109268 | 1:3000 |
| mTOR | Abclonal | A2445 | 1:1000 |
| Bax | Wanleibio | WL01637 | 1:500 |
| Bcl-2 | Wanleibio | WL01556 | 1:500 |
| Caspase-3 | Wanleibio | WL02117 | 1:500 |

Table S2. Antibodies that used in this study
